# Evidence of Filamentary Switching in Oxide-based Memory Devices via Weak Programming and Retention Failure Analysis

**DOI:** 10.1038/srep13599

**Published:** 2015-09-01

**Authors:** Adnan Younis, Dewei Chu, Sean Li

**Affiliations:** 1 School of Materials Science and Engineering, University of New South Wales, Sydney, 2052, NSW, Australia

## Abstract

Further progress in high-performance microelectronic devices relies on the development of novel materials and device architectures. However, the components and designs that are currently in use have reached their physical limits. Intensive research efforts, ranging from device fabrication to performance evaluation, are required to surmount these limitations. In this paper, we demonstrate that the superior bipolar resistive switching characteristics of a CeO_2_:Gd-based memory device can be manipulated by means of UV radiation, serving as a new degree of freedom. Furthermore, the metal oxide-based (CeO_2_:Gd) memory device was found to possess electrical and neuromorphic multifunctionalities. To investigate the underlying switching mechanism of the device, its plasticity behaviour was studied by imposing weak programming conditions. In addition, a short-term to long-term memory transition analogous to the forgetting process in the human brain, which is regarded as a key biological synaptic function for information processing and data storage, was realized. Based on a careful examination of the device’s retention behaviour at elevated temperatures, the filamentary nature of switching in such devices can be understood from a new perspective.

In recent years, resistive random access memories (ReRAMs) have attracted considerable attention because of their remarkable potential for use in non-volatile memories, analogue memristors and neuromorphic applications^1^,^2^. To date, resistive switching (RS) characteristics have been reported in various materials, including transition metal oxides such as CoO^3^, NiO^4^, TiO_2_^5^, and ZnO^6^; perovskites^7^; chalcogenides^8^; organic materials^9^; and even rare-earth oxides^10^. However, in a seemingly parallel line of research, several key challenges still need to be addressed prior to the large-scale manufacturing of ReRAM devices using these materials, including device operational mechanisms, the variation of switching parameters and the limitations on data endurance/retention performance. As many ReRAM materials are strongly correlated electronic/ionic systems, their strong correlation can be exploited in energy-band splitting to tune their electronic band structures and transport properties^11^. Moreover, numerous factors such as stoichiometry, strain and external field (electrical/magnetic) selection can also be adjusted to tailor this electron-ion correlation effect. The many-body mutual interactions among electrons are essential tools for manipulating the RS process, which is an electronic/ionic phenomenon by nature. In the past, various approaches such as current compliance adjustments^11^, voltage sweep rate control^12^, the addition of dopants^13^,^14^ and the introduction of defective layers^15^ have been employed to tune the RS characteristics of various materials. Furthermore, the influence of external fields, such as magnetic fields or light exposure, on RS characteristics has also been studied. Wu *et al.* observed a paramagnetic-to-ferromagnetic phase transition in Mn-doped TiO_2_ thin films by maintaining the conductance of the device in the ON state^16^. Recently, Ungureanu *et al.* effectively utilized illumination to stimulate the RS process in a Pd/Al_2_O_3_/SiO_2_/Si memory cell^17^, where electrons were activated by the light and the Al_2_O_3_ layer acted as an electron-trapping layer. Herein, we present a trapping-layer-free multifunctional RS memory device with a simple structure (Au/CeO_2_:Gd/FTO), whose resistance states can be modulated not only by voltage stimuli but also through ultraviolet (UV) irradiation.

The understanding of the electrical switching mechanisms of RS memories has been limited by the difficulty of characterizing the subtle interactions among chemical, stochastic and localized effects. However, rapid progress in device fabrication and characterization techniques has opened new routes towards the basic understanding and optimization of microscopic memristive switching. Although various mechanisms and models have been proposed^18^,^19^, the conducting filamentary model, which concerns the formation and rupture of conducting bridges/channels, remains the most widely used and most feasible RS mechanism. Recently, high-resolution transmission electron microscopy (HRTEM)^20^,^21^ and conductive atomic force microscopy^7^,^22^ have been used to identify nanoscale conductive filaments and their formation/rupture dynamics, thereby significantly enhancing the understanding of switching mechanisms. However, very few studies have addressed the topics of filament evolution under weak programming conditions and the degradation/dissolution of filaments at elevated temperatures, which are important in the attempt to resolve issues of device reliability. Moreover, weak programming conditions are also very helpful for understanding the inherent learning capabilities (plasticity mechanism) of a device, which are referred to as short-term memory (STM) and long-term memory (LTM). In the STM process, the conductance state rapidly fades away after a weak signal input, whereas the conductance state in LTM is considered to be long-lived and stable. Another important aspect of plasticity behaviour, especially in STM, is that it can serve as an important tool for investigating the volatile nature of the RS process, i.e., the parent conductance state of the device can be retrieved through the elimination of the potential. Additionally, by controlling the input pulse parameters and repetition times, individual states can be achieved, which can help to provide a better understanding of conductive filament growth and the related switching process.

This report presents a systematic examination of the dynamics of conducting filament evolution under continuous weak programming conditions and an investigation of device retention failure at elevated temperatures to elucidate the nature of filamentary switching. The present results provide new insight into the fundamental understanding of switching filament dynamics, which may serve as a basis for computing strategies in neuromorphic engineering and artificial neural networks.

## Results and Discussion

### Current-Voltage Characteristics

**Figure 1:**
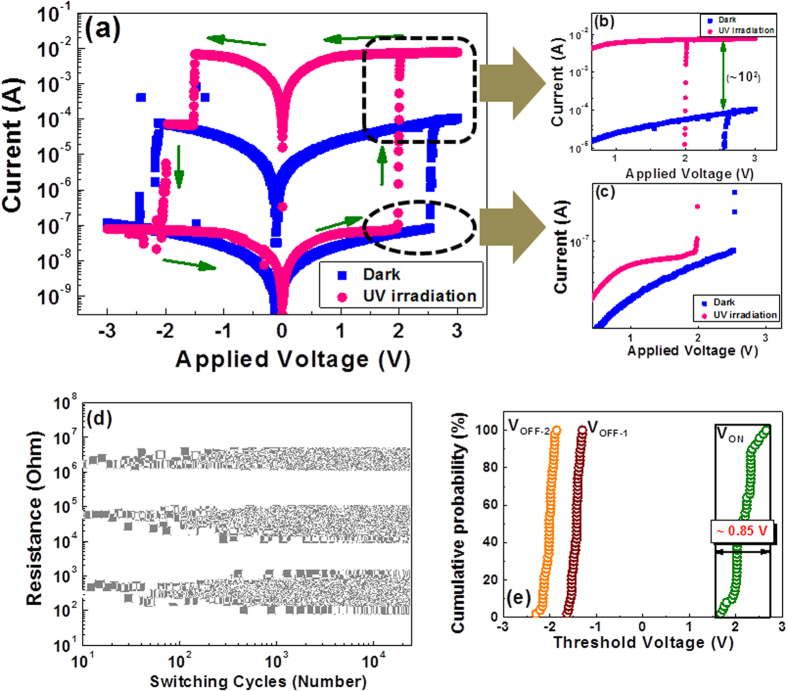
Typical bipolar non-volatile RS characteristics of the CeO_2_:Gd-based memory device. (**a**) I-V curve in semi-logarithmic scale under dark conditions (blue curve) and under UV irradiation (pink curve). (**b–c**) magnified images of low-resistance and high-resistance states. (**d**) Endurance performance of the memory device under UV irradiation over more than 10^4^ switching cycles. (**e**) Distributions of threshold voltages (V_ON_ and V_OFF_) over 50 consecutive switching cycles.

The current-voltage (I–V) behaviour of the pristine Au/CeO_2_:Gd/FTO (GDC) memory device at room temperature (RT) was characterized in dc voltage sweep mode (at a sweep rate of 0.1 Vs^−1^), as shown in Fig. 1(a) (blue curve). With a steady increase in the applied positive potential, a clear transition from the high-resistance state (HRS/OFF) to the low-resistance state (LRS/ON) was observed at approximately 2.56 V; this transition is referred to as the “SET” process. Subsequently, the opposite “RESET” process (at approximately −2.2 V) was also observed when the device was subjected to a negative voltage sweep. Repeating similar measurements under UV irradiation appeared to generate a different device response. The device was set to the LRS at a lower set voltage (~2 V) [pink curve in Fig. 1(a)]. Upon voltage reversal, instead of a single-step reset process, a two-step switching behaviour from the LRS to the HRS was observed. The first transition occurred at V_R1_ (~–1.4 V), after which the resistance of the device briefly remained in an intermediate state until the second transition to the HRS occurred at V_R2_ (~−2 V). Shifts in the conductance of the device in the high- and low-resistance states were also observed, as shown in Fig. 1(b). This shifting of the device threshold voltage to lower values induced by UV irradiation may facilitate the occurrence of a soft breakdown, thus reducing the electric stress required to stimulate the RS effect.

The endurance performance of the GDC memory device under UV radiation was also examined and is shown in Fig. 1(d). All three resistance states (the HRS, the intermediate resistance state (IRS), and the LRS) were well separated for more than 10^4^ consecutive switching cycles. These endurance measurements confirmed that the switching between the ON and OFF states is highly controllable, reversible, and reproducible. The endurance performance of the same device without UV exposure is also shown in Figure S1, which exhibits two conventional resistance states (HRS and LRS); the device maintained these states through 400 switching cycles.

To further verify the reliability of information storage at all three resistance levels and the consistency of the RS characteristics, the same device was subjected to several sequences of SET and RESET processes under irradiation. The device was consecutively switched 50 times between the ON and OFF states, and the distributions of the switching voltage (V_ON_) and reset voltages (V_OFF-1_ and V_OFF-2_) of the device are plotted in Fig. 1(e). Statistical analysis reveals that the range of V_ON_ over 50 cycles was 0.85 V. By contrast, both reset voltages (V_OFF-1_ and V_OFF-2_) exhibited very limited fluctuations over the course of the experiment.

**Figure 2:**
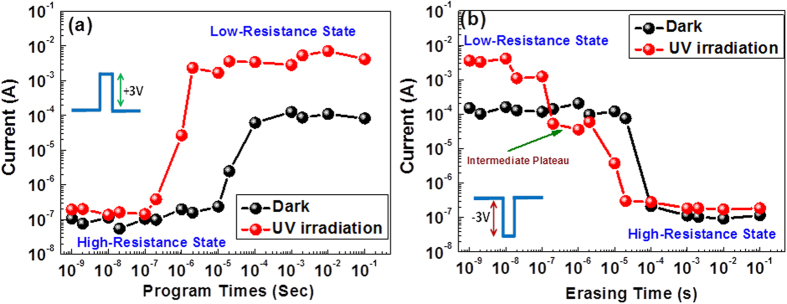
Device performance evaluation based on monitoring of its (**a**) programming and (**b**) erasing characteristics with and without the application of UV irradiation.

The superior device performance observed under UV irradiation was also verified by applying alternating current (AC) pulses to estimate the device response time in the programming and erasing modes. In these measurements, the set and reset characteristics were measured under AC pulse biases with pulse widths ranging from 1 ns to 100 ms at fixed pulse heights of +3/−3 V. A function generator (SRS, SG 390 series) was used to supply the AC pulses of different widths (frequencies), and a source metre (Keithley 2400) was connected to the device to record its current level. To measure the current level of the device, a constant read voltage of 0.3 V was applied for 3 seconds with a step of 0.1 s. The values shown in Fig. 2(a) represent the average current values recorded between 0.5 and 3 s during the application of the read voltage. Moreover, the same measurements were repeated at different times for three different samples. The current values presented in Fig. 2 are the average set and reset times for the three different samples.

During the programming test, the device switched from the HRS to the LRS at +3 V/2 μs under irradiation, whereas a longer period of time was required to switch the device from the OFF state to the ON state when it was operated under normal conditions (+3 V/100 μs), as shown in Fig. 2(a).

Similar behaviour, namely, a relatively fast response time, was observed in negative voltage pulse mode, in which the resistance state of the device switched from low to high at −3 V/100 μs [Fig. 2(b)]. Interestingly, under UV irradiation, the device again exhibited multi-step reset behaviour; it first underwent to an intermediate plateau at −3 V/0.2 μs and then returned to its initial HRS at −3 V/20 μs, as shown in Fig. 2(b).

Currently, the electrochemical redox process associated with the formation and annihilation of conductive filaments, which predominantly consist of oxygen vacancies (V_o_)^19^,^23^, is regarded as the most plausible and widely accepted RS switching mechanism. However, clear visualization of the conducting filaments in insulating oxides remains a considerable challenge^24^. To investigate the filamentary switching mechanism in these devices, several GDC samples with Au top electrodes of various diameters (50, 75, 100 and 250 μm) were fabricated to determine the ON and OFF states, as shown in Figure S2. The resistance ratios (R_Off_/R_On_) were found to be nearly inversely proportional to the size of the top electrode. It is reasonable to conclude that the switching mechanism of this device is based on filamentary switching, which affects only an extremely small portion of the entire electrode area; the remaining electrode area contributes a (non-switching) parallel resistance. As a consequence, the R_Off_/R_On_ ratio significantly improved when the electrode area was reduced, hence evidencing the filamentary switching mechanism in these devices^19^.

### Weak Programming Characteristics

**Figure 3:**
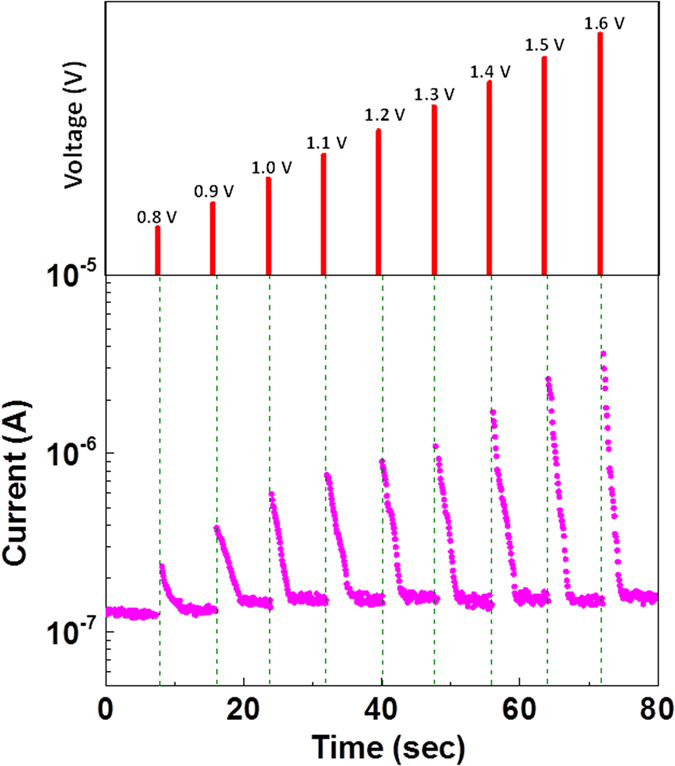
Volatile and non-volatile memory performance of a CeO_2_:Gd device. The current change was observed by applying a sequence of positive voltage pulses with a width of 0.1 s at intervals of 8 seconds s. The read voltage was 0.1 V.

To obtain useful insights on the underlying switching mechanism of the pristine device, weak programming conditions, i.e., a sequence of low-amplitude input voltage pulses (0.8 V to 1.6 V (pulse widths of 0.1 s) followed by a continuous read potential (0.1 V) for 8 seconds, were imposed to observe the current-fading process. In response to each input voltage pulse, the instantaneous current was observed to gradually increase with increasing amplitude of the applied voltage pulses, as shown in Fig. 3. This interesting behaviour originated from the unique migration process of oxygen/oxygen vacancies under voltage stimuli. The current decay process that was observed after every positive input voltage pulse is attributed to the diffusion of weakly bonded oxygen from the bulk region towards the Au top electrode (TE) along an electrochemical potential gradient. The current augmentation that resulted from the increase in the pulse amplitude may be attributable to a local Joule heating effect facilitating the oxygen migration process. The presence of intrinsic and weakly bonded oxygen species in the pristine device (immediately after UV exposure) was verified by X-ray photospectroscopy (XPS), as shown in the Supplementary information Figure S3. The O 1s peaks could be fitted with three peaks attributed to lattice oxygen at approximately 529.3–530.0 eV (O_A_), chemisorbed oxygen and/or weakly bonded oxygen species at approximately 531.3–531.9 eV (O_B_), and surface oxygen in hydroxyls and/or surface-adsorbed water at approximately 532.6 eV (O_C_)^25^,^26^. The superposition of weak programming pulses could induce a local diffusion/migration process among weakly bonded oxygen species within the device, which yielded useful insights into the device’s plasticity behaviour.

A transition in device behaviour from volatile memory to non-volatile memory, which is analogous to the transition from the STM to LTM (plasticity behaviour) in the human brain, can be realized by imposing weak programming conditions. Such plasticity behaviour, created by weak stimulation, can result in the exponential decay of STM and ultimately lead to complete erasure^27^,^28^. Moreover, by gradually increasing the amplitude of the voltage pulses, the device memorization power can be incrementally increased. Once the device undergoes a formation process by passing the threshold potential (set voltage), non-volatile memorization can be achieved, as shown in Fig. 1(a).

**Figure 4:**
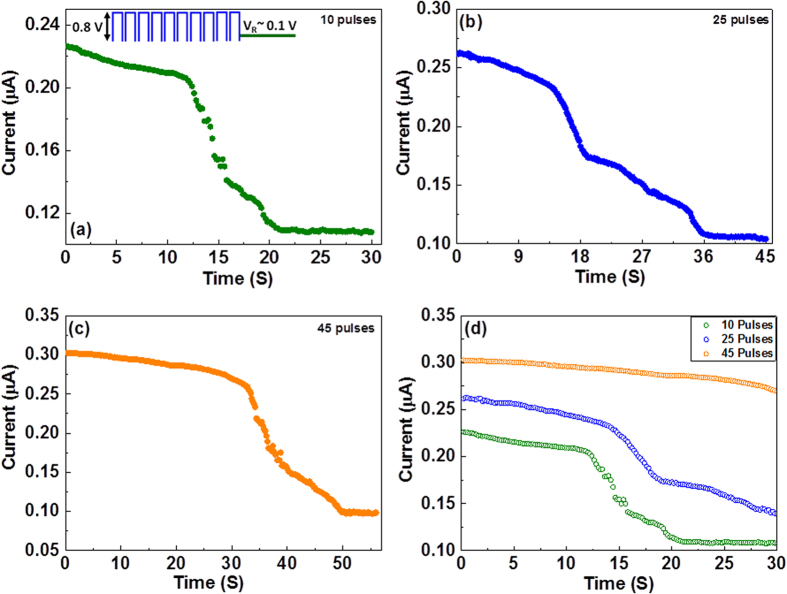
Measurement of pulse relaxation behaviour over a certain period of time. Bursts of varying numbers of pulses (0.8 V) (**a**) 10 pulses, (**b**) 25 pulses, and (**c**) 45 pulses — were imposed on the memory device to induce potentiation, and the corresponding current relaxation times were measured at a low read voltage of 0.1 V. (**d**) The same curves re-plotted on a 30 s time scale to emphasize the short-term to long-term memory transformation.

The phenomenon of STM corresponds to a neuronally induced synaptic weight modification that tends to relax towards a resting state, thereby giving rise to activity-dependent signal processing^29^. To further evaluate the plasticity behaviour of our device, continuous weak programming signals were implemented. The device was exposed to a series of pulses of fixed amplitude (0.8 V) and width (10 mS) to modulate the potentiation; this was followed by a short read voltage to examine the device relaxation behaviour, as shown in Fig. 4.

The results obtained from this measurement coincide well with the weak pulse measurements presented in Fig. 3. During the measurement, the device required only a short relaxation time to return to its original state. In other words, reduced stability was observed in a low ON state due to the relatively thin filaments [Fig. 4(a)]. Exposing the device to a large number of voltage pulses [Fig. 4(b)] caused the conductance state to shift to higher levels, thus increasing the relaxation time constant. In essence, thick and stable filaments formed as a consequence of the large number of pulses (as evidenced by the increase in conductance), and therefore, these filaments required more time to dissolve/annihilate. To achieve a better understanding of the effects of the pulse characteristics on the relaxation time, the conductance states of the device and the corresponding relaxation times under three different conditions (10, 25 and 45 pulses) were re-plotted on a time scale of 30 seconds, as shown in Fig. 4(d). This figure reveals that the device conductance values could be effectively tuned by simply controlling the programming conditions. The lowest conductance led to STM (complete relaxation within 30 seconds), and the highest conductance may correspond to LTM (minimal relaxation within 30 seconds). To ensure device reproducibility, these measurements were repeated on five different samples, and their relaxation behaviours after the application of different numbers of identical voltage stimuli were recorded. All devices exhibited similar behaviour: the conductance (current level) of each device was initially enhanced and later decayed to its inherent level, exhibiting a short relaxation time (~15 to 20 s) in the case of fewer pulses and a relatively long relaxation time (~50 to 60 s) in the case of more pulses. This behaviour was reproducible in three independent sets of measurements performed on each sample.

Based on the aforementioned experimental results, it is reasonable to conclude that the switching mechanism of these devices is related to the evolution/rupture of conducting filaments.

### Retention Failure Analysis

**Figure 5:**
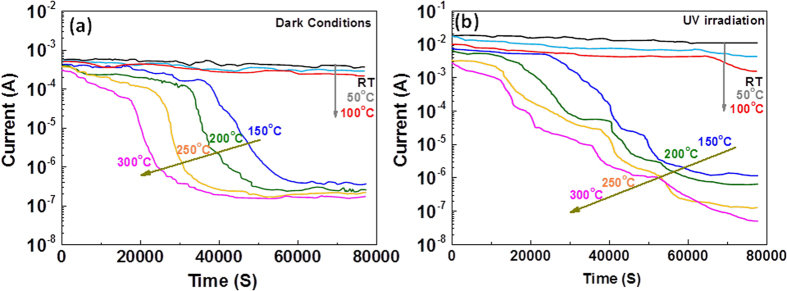
Evidence of the filament dissolution process through measurements of the temperature-dependant retention versus time. The device exhibited excellent data retention up to 100 °C when operating under both dark and irradiated conditions. However, data retention failure was clearly observed at elevated temperatures both (**a**) under dark conditions and (**b**) under UV irradiation. A read pulse (0.1 V/10 ms) was applied every 5 s during the test.

To further confirm the aforementioned filamentary switching mechanism, the temperature dependence of the switching behaviours was studied to gain insight into the nature of filamentary failure. The device conductance in the LRS was periodically monitored every 10 s with a low read voltage pulse (0.1 V/10  ms) to avoid disturbance in the the device state at elevated temperatures (RT, 50, 100, 150, 200, 250 and 300  °C), as shown in Fig. 5. The device retained its data retention capability up to 100 °C for more than 24 hours, thereby demonstrating excellent thermal stability and sustainability. At higher temperatures, the failure of the device was more rapid than that at low temperatures [Fig. 5(a)]. In general, two different conductance behaviours were recorded at temperatures higher than 100  °C, and a gradual decline in retention was observed prior to complete failure. Our previous results are consistent with the observed high-temperature retention failure phenomena, suggesting that the entire process can be understood in terms of the oxygen ions/vacancies-based filament model. Generally, when a sufficient number of oxygen vacancies become aggregated or accumulated inside a filament, a percolation path in the form of a conducting filament is generated, resulting in the LRS. However, the oxygen vacancies inside the filament can also spontaneously diffuse away via a thermally activated process^30^. To understand this process, the retention failure time was recorded and analysed as a function of temperature, as shown in Figure S4, which exhibits a thermal activation effect with an activation energy of 0.6 eV, in good agreement with the thermal activation energy of bulk CeO_2_^31^. The gradual reduction in the concentration of oxygen vacancies in the filament through diffusion corresponds to the gradual decline in the retention failure curve. Finally, once the local concentration of oxygen vacancies inside the filament has decreased below a certain threshold value, i.e., the electron wave functions associated with the oxygen vacancies no longer overlap and no extended state remains^32^,^33^, the filament ultimately ruptures, resulting in sudden decrease in device conductance.

The device exhibited similar behaviour while operating under UV irradiation, as shown in Fig. 5(b). Despite the observation of regions of both gradual and abrupt decline in the retention test, evidence for the rupture of multiple filaments could also be observed at higher temperatures (150 °C and above). After the first abrupt drop in conductance at ~ 40,000 s, the conductance reached an intermediate plateau at ~55,000  s before eventually returning to the background level after a second decline at ~60,000  s.

**Figure 6:**
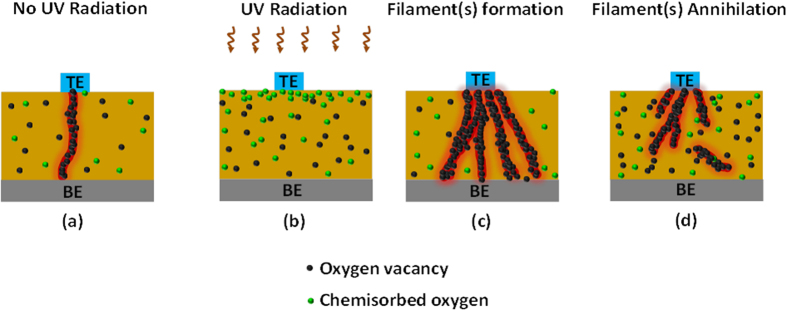
The generation and annihilation of conducting filaments under various conditions. (**a**) The formation of a single filament, in the absence of UV exposure, under an applied positive potential. (**b**) UV exposure causes an increase in the O_B_ concentration on or near the GDC film surface. (**c**) The formation of multiple conducting filaments under the combination of UV irradiation and a positive potential, increasing the current level of the low-resistance state (**d**) The annihilation/rupture of conducting filaments as a consequence of an applied negative potential.

GDC nanocrystals are generally considered to have many surface defects, such as oxygen vacancies that can interact with oxygen molecules in the atmosphere. These interactions can generate various intermediates, such as superoxide (O_2_^−^), peroxide (O_2_^2−^) and O^−^ species, before reducing these intermediates to lattice oxygen (O^2−^)^34^. It is believed that UV radiation can preferentially facilitate the formation of superoxides (chemically adsorbed oxygen) through the reaction of oxygen molecules with electrons trapped on the surface^35^. To verify this hypothesis, XPS studies were conducted using the same GDC sample without and with UV radiation exposure, as presented in Figures S5 and S3, respectively. UV exposure was found to facilitate the formation of chemisorbed oxygen (O_B_). Under the application of a suitable potential, O_B_ can be attracted towards the top electrode through Columbic interactions. This results in the evolution of a conducting filament and enhances device conductance. With a further increase in the applied potential, the increased O_B_ migration towards the anode generates more oxygen vacancies at a proportional concentration. As a result of this migration, conducting filaments consisting of oxygen vacancies grow between two electrodes. In the case of device operation without UV exposure, the relatively low O_B_ concentration is likely to result in a small or weakly conducting filament between the two electrodes. By contrast, under UV exposure, the higher O_B_ concentration, likely facilitates the formation of one strong filament or multiple filaments between the cathode and the anode. As a consequence, an elevated current level was observed for the device in the LRS [Fig. 1(a)], along with much more rapid response times for programming and erasing [Fig. 2(a)]. The proposed mechanism of filament formation and annihilation in the absence or presence of UV irradiation is schematically illustrated in Fig. 6.

## Summary

We report the multifunctional phenomena of bipolar non-volatile resistive switching and volatile rectification in a two-terminal CeO_2_:Gd-based memory device. Such phenomena can be achieved through control of the local migration of oxygen ions/vacancies by means of UV irradiation. The plasticity behaviour of the memory device was also investigated. A transition from short-term to long-term memory can be achieved by adjusting the excitation strength (number of pulses) to control the maximum conductance over a given period of time. Furthermore, the oxygen ion/vacancy-mediated filamentary switching behaviour was verified by measuring the temperature dependence of the retention failure time. The resulting comprehensive understanding of the short-term to long-term memory transition and failure mechanisms of this oxide-based memory device provides new fundamental insights for the improvement of device performance and the investigation of underlying switching mechanism. The present work may also suggest a new route for the development of circuits, analogue memories and artificial neural networks.

## Methods

The growth of CeO_2_:Gd on fluorine-doped tin oxide (FTO, 9.3 ~ 9.7 Ω, Asahi Glass Corporation, Japan, 1.1 mm × 26 mm × 30 mm) was carried out using an electrochemical deposition process with an aqueous solution containing 0.01 M Ce(NO_3_)_3_.6H_2_O, 0.01 M Gd(NO_3_)_3_.9H_2_O, 0.05 M NH_4_Cl and 0.05 M KCl with a current density of 0.5 mA/cm^2^ for 2 hours at 70 °C using an Autolab 302N Potentiostat. A standard three-electrode setup in an undivided cell was used. The FTO substrate was used as the working electrode, and platinum foil (0.2 mm × 10 mm × 20 mm) was used as the counter electrode. The distance between the two electrodes was 30 mm. The reference electrode was an Ag/AgCl electrode in a 3 M KCl solution, against which all potentials reported herein were measured.

X-ray photoelectron spectroscopy (XPS) was performed using an ESCALAB 250Xi spectrometer with a monochromatized Al K-alpha X-ray source (*h*ν = 1486.6 eV) with a 20 eV pass energy.

The composition of Gd:CeO_2_ (GDC) is CeO_2_ doped with 5 at% Gd^3+^. To study the crystal structure of the as-synthesized GDC material, X-ray diffraction studies were performed, and the recorded diffraction pattern is illustrated in Figure S6. All observed peaks correspond to the CeO_2_ (111), (200), (220), and (311) planes, indicating that the material can be indexed as a face-centred cubic phase of the space group *Fm3m*, as identified using the standard JCPDS card 34–0394.

To measure the electrical properties (resistive switching characteristics) of the films, circular Au top electrodes of approximately 250 μm in diameter were patterned and deposited via sputtering using a metal mask. The current-voltage curves of the devices were recorded (at a voltage sweep rate of 0.1 Vs^−1^) using an Autolab 302N electrochemical workstation controlled with Nova software. For the pulse measurements, a function generator (SRS, SG 390 series) was used to deliver AC pulses of different widths (frequencies), and a source metre (Keithley 2400) was connected to the device to record its current level (at a 0.3 V read voltage). A table-top UV/ozone surface processor (SSP16-110, with a maximum power of 95 W and a wavelength of 254 nm) was used to perform electrical measurements under UV irradiation. Moreover, the UV chamber was equipped with a built-in lamp cooling system (operated via forced-air cooling and fitted with a blower), which maintained a constant temperature (within +/−2 degrees of room temperature) inside the UV chamber. All measurements were performed at room temperature unless otherwise indicated.

## Additional Information

**How to cite this article**: Younis, A. *et al.* Evidence of Filamentary Switching in Oxide-based Memory Devices via Weak Programming and Retention Failure Analysis. *Sci. Rep.* **5**, 13599; doi: 10.1038/srep13599 (2015).

## Supplementary Material

Supplementary Information
