# Supplementary material for: Evidence of Filamentary Switching in Oxide-based Memory Devices via Weak Programming and Retention Failure Analysis
Source: Sci Rep. 2015 Sep 1;5:13599. doi: 10.1038/srep13599 (PMC4555098; doi:10.1038/srep13599)
Supplement: Supplementary Information [file srep13599-s1.pdf]

Evidence of Filamentary Switching in Oxide-based Memory Devices via Weak Programming and Retention Failure Analysis

Adnan Younis*, Dewei Chu* and Sean Li

School of Materials Science and Engineering, University of New South Wales, Sydney, 2052, NSW, Australia

Email: a.younis@unsw.edu.au, d.chu@unsw.edu.au

Supporting Information

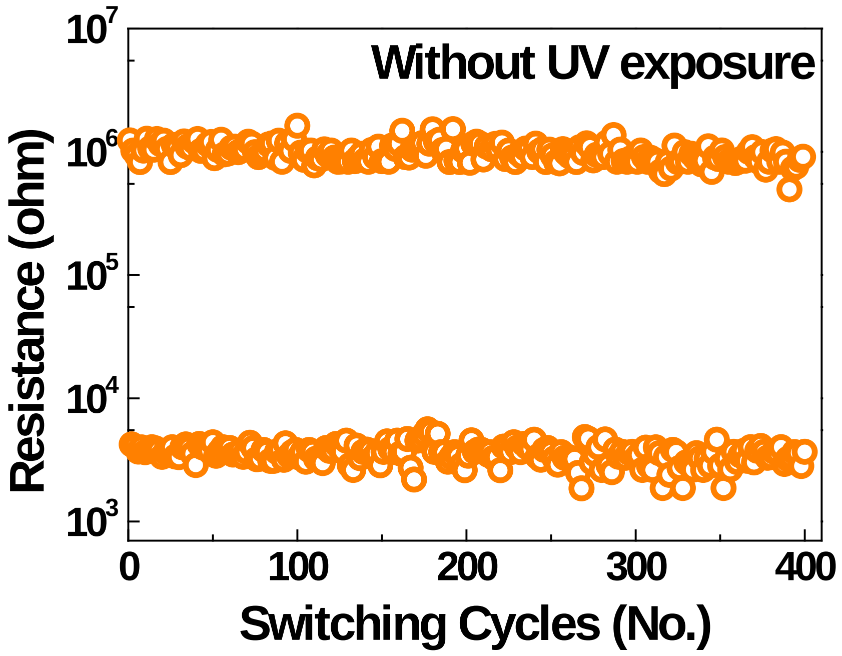

Figure S1: Data endurance performances of CeO_2:Gd under normal condition.

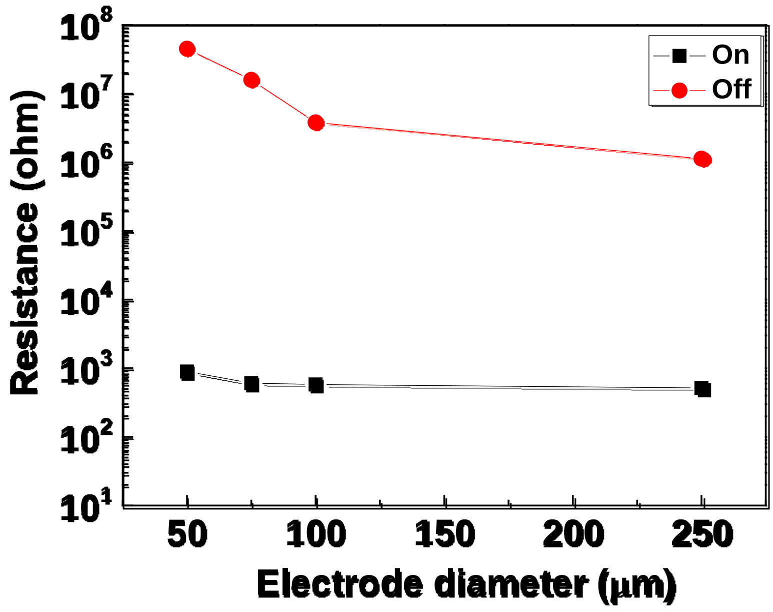

Figure S2: Area dependence of resistance values in the ON and OFF states for GDC memory cells.

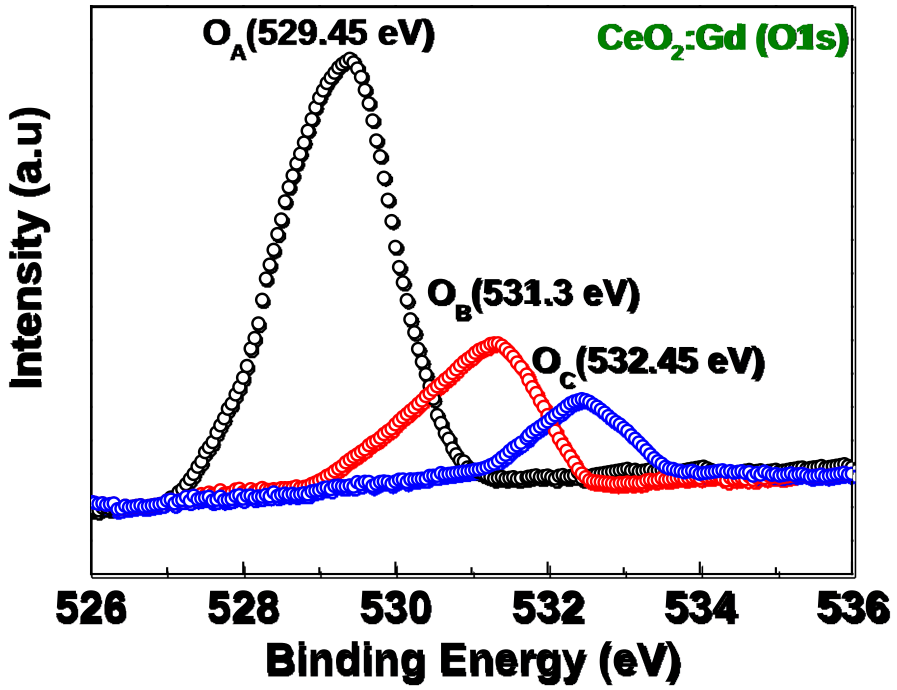

Figure S3: X-ray photoelectron spectroscopy data for O1s taken from CeO_2:Gd sample immediately exposed after UV radiation. The peaks indicate Gaussian fits with peaks at 529.45, 531.3 and 532.45eV.

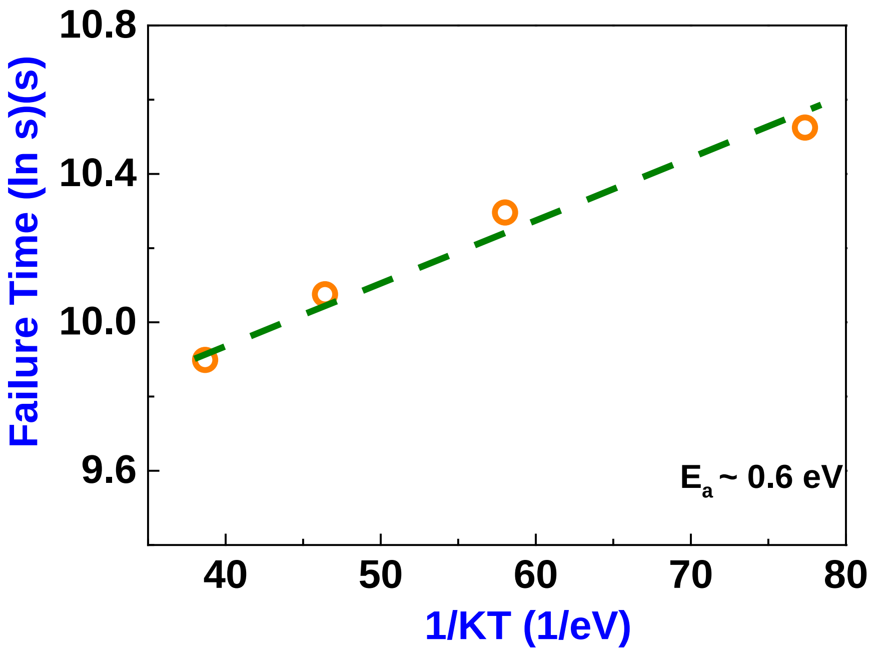

Figure S4: Temperature dependence of the characteristics retention failure time (circles) and fitting (line) following the Arrhenius equation.

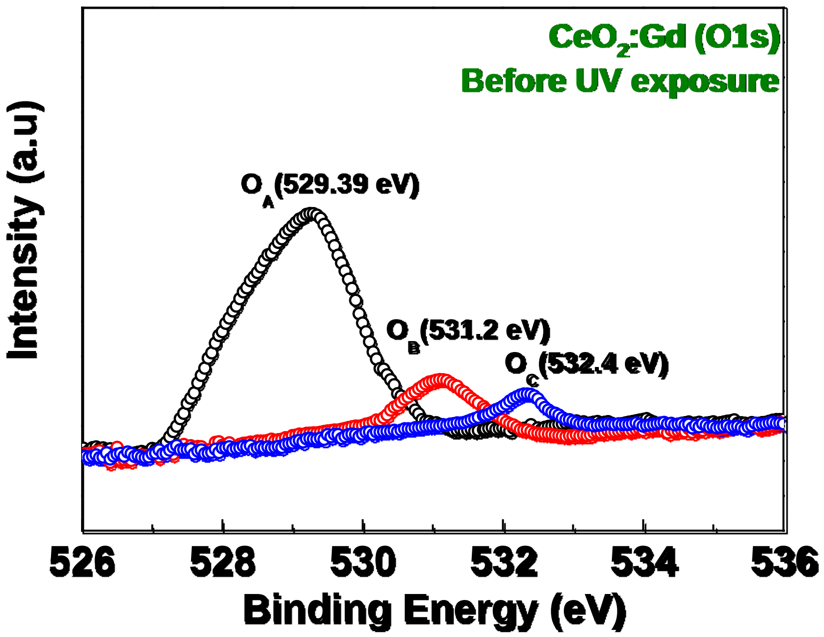

Figure S5: X-ray photoelectron spectroscopy data for O1s (a) without UV exposure and (b) immediately after UV exposure.

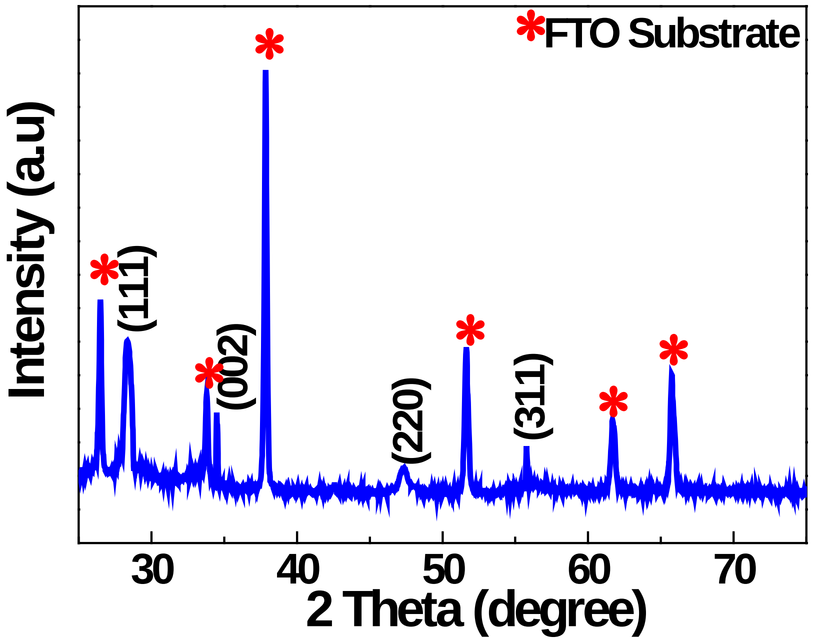

Figure S6: (a) X-ray diffraction pattern of as-prepared GDC film.
